# Scale to Assess Empowering Nurse Leader Communication Behaviours the Turkish Version Validity and Reliability: A Methodological Study

**DOI:** 10.1002/nop2.70588

**Published:** 2026-05-10

**Authors:** Sultan Türkmen Keskin, Meltem Özduyan Kiliç, Ayten Demir

**Affiliations:** ^1^ Department of Nursing, Faculty of Nursing Ankara University Altındağ /Ankara Turkey

**Keywords:** communication, empowerment, nurses, scale development

## Abstract

**Aim:**

This study examines the validity and reliability of the Scale to Assess Empowering Nurse Leader Communication Behaviours when adapted to Turkish.

**Design:**

The research was designed as a methodological study.

**Method:**

This study was conducted in a university hospital in Ankara between February and May 2024. Analyses were conducted using SPSS 25 and AMOS 24. Construct validity was assessed using an Exploratory Factor Analysis with a sample consisting of 280 nurses and a Confirmatory Factor Analysis with a sample also consisting of 280 nurses. Internal consistency and stability were assessed using Cronbach's alpha and the intraclass correlation coefficient (ICC).

**Results:**

Content validity index (CVI) was 0.833. The KMO sample adequacy was 0.942, and Bartlett's test yielded *χ* ^2^(253) = 4398.160 (*p* < 0.001). The model explained 57.8% of the total variance, and the fit indices indicated a good fit. The Empowering and Limiting sub‐dimensions showed Cronbach's alphas of 0.943 and 0.883, respectively, and intraclass correlation coefficients (ICCs) of 0.868 and 0.932, respectively. While the original two‐factor structure (empowering and limiting communication) was preserved in the Turkish adaptation, the number of items was reduced from 35 to 20.

**Conclusion:**

This scale can be used to assess empowering communication behaviours among nurse managers in Turkish society.

**Reporting Method:**

The research methodology adhered to the STROBE guidelines for epidemiological studies.

**Patient or Public Contribution:**

No patient or public contribution.

## 1 Introduction

In nursing, effective leaders are responsible for creating supportive and empowering working conditions to ensure safe, high‐quality care (Hopkinson et al. 2021). In fulfilling this responsibility, they manage a multidirectional communication process that encompasses colleagues, patients, and other healthcare professionals. The American Organization for Nursing Leadership (AONL) described “Communication and Relationship Building” as one of the five core competencies that nurse managers need to have in its Nurse Leader Competency Model (AONL 2022). This core competency is a key factor in the effective management of health services. Nurse managers who possess this competency improve patient care outcomes (Fowler et al. 2021; Jankelová and Joniaková 2021) and support nurses' empowerment by fostering a positive working environment (Cho et al. 2021; Hopkinson et al. 2021). However, these management and communication styles are not universal and can differ depending on cultural and contextual factors.

Empowerment refers to the individual's sense of being able to complete their work in a meaningful and successful way (Laschinger et al. 2010). It is important to create positive structural and psychological conditions that empower nurses (Gottlieb et al. 2021). Structural empowerment entails providing nurses with access to the resources, information, support, and opportunities necessary for them to do their jobs effectively (Dan et al. 2018; Gottlieb et al. 2021; Laschinger et al. 2010). Psychological empowerment, on the other hand, is the development of a sense of professional determination and competence along with the perception of meaning and value that the nurse attributes to his/her job (Laschinger et al. 2010; Shapira‐Lishchinsky and Benoliel 2019). Given that empowerment is shaped not only by organizational structures but also by cultural norms surrounding authority, hierarchy, and communication, understanding how nurse leaders communicate in different sociocultural settings is particularly important.

The fact that nurse managers share information on time, communicate important issues, ask the nurses they work with for their opinion, are understandable, good listeners, communicate bidirectionally, and are transparent in their thoughts and discourses is what empowers nurses (Hopkinson et al. 2019, 2021; Laschinger et al. 2010). Nurses who feel empowered are more committed to their institution (Albasal et al. 2022; Rawah and Banakhar 2022); they do not wish to leave (Huang et al. 2024), have greater job satisfaction, and show better performance (Choi and Kim 2019; Saleh et al. 2022). In addition, nurses can act more independently in their professional practices and participate more actively in decision‐making (Gottlieb et al. 2021). This, in turn, helps to achieve quality patient outcomes (Fiorini et al. 2022). Therefore, the effectiveness of formal communication practices is important. How empowering communication behaviours are articulated, interpreted, and evaluated may differ across healthcare systems with varying hierarchical structures and power distributions.

Communication errors in healthcare negatively affect patient safety (Kämäräinen et al. 2024) and are often rooted in hierarchical organizational cultures that may reinforce employee silence (Lee and Lee 2024; Lee et al. 2025; Kepplinger et al. 2024). In hierarchical cultures, where decision‐making authority rests with upper management, nurses may perceive their input as ineffective or meaningless. This perception reinforces the belief that voicing concerns will not lead to meaningful change, ultimately resulting in the suppression of critical information for patient safety (Lee and Lee 2024). Within these hierarchical cultures, the attitudes and communication styles of nurse managers are particularly influential in either perpetuating silence or fostering a climate in which nurses feel safe to speak up (Krenz et al. 2020; Lee and Lee 2024; Lee et al. 2025). Consequently, nurse managers, whose primary responsibility is to oversee and coordinate care, must develop and enact empowering communication behaviours that reduce perceived risks and actively promote open, bidirectional dialogue.

The “Scale to Assess Empowering Nurse Leader Communication Behaviours,” developed by Hopkinson et al. (2021), focuses on nurse leaders' communication and its impact on nurse empowerment. Although several communication assessment tools exist in Türkiye (Buluş et al. 2017; Mendi et al. 2020), no scale specifically assesses the empowering communication behaviours of nurse leaders. Therefore, this study aimed to adapt the “Scale to Assess Empowering Nurse Leader Communication Behaviours” to Turkish culture and to examine its validity and reliability in Turkish. By providing a culturally adapted measurement scale, this study contributes to the international nursing literature on leadership and empowerment while enabling cross‐cultural comparisons of empowering communication behaviours among nurse leaders.

### 1.1 Research Question

- Is the Turkish version of the Scale to Assess Empowering Nurse Leader Communication Behaviours suitable for use in Turkish culture?

## 2 Methods

### 2.1 Study Design

This methodological study assessed the psychometric properties of the Scale to Assess Empowering Nurse Leader Communication Behaviours in Turkish. This study first verified linguistic equivalence to the original using the translation‐back‐translation method. Then, ten experts assessed the scale's content validity. Subsequently, validity and reliability analyses were conducted, and the scale was finalized accordingly. The research methodology was carried out in accordance with the STROBE guidelines for observational epidemiological studies.

### 2.2 Translation Procedure

The translation‐backtranslation method was used to ensure the scale's language equivalence (Hambleton and Patsula 1998). Two linguists translated the measurement tool from English into Turkish. The scale was given its initial Turkish form taking into account the similarities and the differences between the two translations. The scale was then presented to a Turkish Language expert for evaluation of its compatibility with Turkish. Suggested corrections were made in line with the language expert's comments. Another expert then translated the Turkish scale back into English to determine whether it matched the original version. To ensure language equivalence, the translated version was mailed to the original author for review. The author confirmed the linguistic equivalence of the translation.

A pilot study was conducted to check the comprehensibility of the scale items and their suitability for the language. The pilot study was concluded after the first 10 participants, as no suggestions or revisions were required. No additional adjustments were made to the scale items.

### 2.3 Participants and Setting

The research was conducted at a large tertiary university hospital in Ankara, Türkiye. This institution, with approximately 1000 registered beds and 886 active nurses, provides complex and advanced treatment services. The hospital has a broad and multidisciplinary structure encompassing inpatient wards that provide services across numerous internal medicine and surgical specialties, operating rooms, an emergency department, intensive care units, chemotherapy units, and a variety of outpatient clinics. The study included nurses who were actively working in all inpatient and outpatient units of the institution (such as medical and surgical wards, operating rooms, outpatient clinics, emergency departments, intensive care units, and chemotherapy units), who were able to speak and understand Turkish, and who voluntarily agreed to participate in the study. Nurses working in a managerial role (e.g., director of nursing services, chief nurse, or charge nurse) were excluded.

The literature recommends achieving a sample size of 5–10 times the number of items in the scales (Alpar 2022; DeVellis 2017). Accordingly, 560 participants were used to adapt the 35 items. In the retest phase of the measurement tool, the scale was readministered to 30 of these participants 2 weeks later. Two independent samples were used for exploratory and confirmatory factor analyses.

### 2.4 Data Collection Tools

The data for the study were collected using the demographic questionnaire and the Scale to Assess Empowering Nurse Leader Communication Behaviours.

#### 2.4.1 Demographic Questionnaire

This demographic information form, developed by researchers, consists of seven items designed to identify participants' personal and professional characteristics. The form includes variables such as gender, age, and educational level, as well as professional factors including the current working unit and total years of experience in the nursing profession.

#### 2.4.2 The Scale to Assess Empowering Nurse Leader Communication Behaviours

The Scale to Assess Empowering Nurse Leader Communication Behaviours was developed by Hopkinson et al. in 2021. The scale is used to evaluate nurse managers' communication behaviours. The scale comprises eight conceptual components (comprehensibility, manner, listening, openness, feedback, empathy, nonverbal communication, and paralanguage) and is structured into two sub‐dimensions. The Empowering sub‐dimension consists of 20 positive items (Cronbach's *α* = 0.972), whereas the Limiting sub‐dimension consists of 15 negative items (Cronbach's *α* = 0.935).

The Empowering sub‐dimension includes items 1, 2, 6, 12, 14, 15, 16, 17, 18, 20, 21, 22, 23, 24, 25, 26, 27, 28, 29, and 32, while the Limiting sub‐dimension includes items 3, 4, 5, 7, 8, 9, 10, 11, 13, 19, 30, 31, 33, 34, and 35. The scale items are scored using a 5‐point Likert‐type scale ranging from 0 (never) to 4 (always). The mean scores for the two sub‐dimensions are calculated and evaluated separately. A higher mean score in the empowering sub‐dimension relative to the limiting sub‐dimension indicates that nurse leaders exhibit empowering communication behaviours more frequently.

### 2.5 Data Collection

Data collection was conducted by researchers through face‐to‐face interviews between February and May 2024. Prior to data collection, participants were informed about the study's objectives, and the questionnaire was administered only to those who provided voluntary consent. To assess test–retest reliability, the measurement scale was re‐administered to a subgroup of 30 participants 2 weeks after the initial assessment. During the study period, approximately 600 nurses were approached, of whom 560 agreed to participate.

### 2.6 Statistical Analyses

The researchers used IBM SPSS 25 and AMOS 24 to analyse the research data. Demographic characteristics were summarized using descriptive statistics. Various methods were used to evaluate the scale's validity and reliability. Exploratory and confirmatory factor analyses (EFA, CFA) were performed to determine construct validity. Using different data sets is recommended to reliably verify the factor structure in scale adaptation studies (Brown 2015; Kalkbrenner 2021). For factor analyses, the study sample (*N* = 560) was randomly divided into two equal groups: one for exploratory analysis and another for confirmatory analysis (*n* = 280 each). In determining the sample size in methodological studies, the number of participants is recommended to be at least five times the number of items for factor analysis (Alpar 2022; DeVellis 2017). According to these criteria, the sample size of the current study is sufficient for statistical analysis. Since there was a relationship among the adapted scale sub‐factors, Principal Axis Factoring and Promax rotation were preferred for EFA (Field 2024). Factors with eigenvalues ≥ 1 were considered when determining the number of factors. Data suitability for factor analysis was assessed using the Kaiser‐Meyer‐Olkin (KMO ≥ 0.70) and Bartlett's test of sphericity (*p* < 0.05; Field 2024; George and Mallery 2024).

Confirmatory factor analysis (CFA) was used to assess the adequacy of scale items in measuring the predicted structure. Model fit was evaluated using the ratio of chi‐square to degrees of freedom (*χ* ^2^/df) less than 3 and the root mean square error of approximation (RMSEA) less than 0.08, which were set as the basic criteria for acceptable fit. In addition, values for the Standardized Root Mean Square Residual (SRMR) < 0.05, and values ≥ 0.90 for the Goodness of Fit Index (GFI), Comparative Fit Index (CFI), Tucker‐Lewis Index (TLI), Non‐Normed Fit Index (NFI), and Incremental Fit Index (IFI) were considered acceptable fit criteria (Hair et al. 2019; Kalkbrenner 2021).

Composite Reliability (CR) and Average Variance Extracted (AVE) were calculated to assess convergent validity. As for the scale's reliability analyses, item‐total score correlations, internal consistency (Cronbach's *α*), split‐half reliability, and a discriminant analysis were conducted on the upper and lower 27% groups. The intraclass correlation coefficient (ICC) was used in the test–retest analysis for reliability over time.

### 2.7 Ethical Considerations

Permission was obtained from the principal author for the use of the Scale to Assess Empowering Communication Behaviours of Nurse Leader. Approval was obtained from the Ankara University Ethics Committee (Date: September 13, 2023; number: E‐64446339‐020‐154252). This study was conducted in accordance with the Declaration of Helsinki (as revised in Brazil, 2013). Administrative approval was obtained from the institution where the research was conducted. Each participant signed an informed consent document prior to the face‐to‐face interview.

## 3 Results

### 3.1 Sample Characteristics

Of the nurses participating in the study (*n* = 560), 36.4% were aged 22–29, 86.8% were women, and 82.7% had an undergraduate degree. In terms of professional experience, 45.5% had 11 or more years, and 39.5% had worked 11 or more years at their hospital. 40.4% of the nurses had been working on their current units for 1–5 years, and 82.1% work on adult units (Table 1).

**TABLE 1:** Descriptive statistics for the socio‐demographic characteristics of nurses (*n* = 560).

Variables	Subgroups	Number (*n*)	Percentage (%)
Age	22–29	204	36.4
	30–34	128	22.9
	35–44	125	22.3
	45 and over	103	18.4
Gender	Female	486	86.8
	Male	74	13.2
Education level	Medical vocational high school/associate degree	41	7.3
	Undergraduate	463	82.7
	Post‐Grad and Ph.D.	56	10.0
Years of professional experience as a nurse	Less than 1 year	25	4.5
	1–5 years	162	28.9
	6–10 years	118	21.1
	11 years or more	255	45.5
Years of experience in the current hospital	Less than 1 year	40	7.1
	1–5 years	198	35.4
	6–10 years	101	18.0
	11 years or more	221	39.5
Years of experience in the current unit	Less than 1 year	69	12.3
	1–5 years	226	40.4
	6 to 10 years	98	17.5
	11 years and more	196	29.8
Unit	Adult unit	460	82.1
	Paediatric unit	74	13.2
	Other	26	4.7

### 3.2 Content Validity

Opinions from 10 experts were collected to assess the scale's content validity. The expert panel consisted of two clinical nurses with more than 8 years of experience in the Paediatric Surgery and Internal Medicine clinics; two nurse managers with over 10 years of leadership experience (a director of nursing services and a charge nurse); and six academics, three specializing in Psychiatric Nursing and three in Nursing Management. All academics held doctoral degrees and had between one and 5 years of clinical experience. The Davis technique was used to determine content validity. Experts rated each item on a 4‐point scale: 1 (not appropriate), 2 (needs major revision), 3 (needs minor revision), and 4 (very appropriate). The content validity index (CVI) was calculated by dividing the number of experts rating the item as 3 or 4 by the total number of experts (Davis 1992; Gültürk 2024). According to expert assessments, the scale's average content validity index (CVI) is 0.833.

### 3.3 Construct Validity

The construct validity of the scale was assessed through Exploratory Factor Analysis (EFA) and Confirmatory Factor Analysis (CFA). The adequacy of the sample size for factor analysis was assessed using the Kaiser‐Meyer‐Olkin (KMO) test. Consequently, KMO was calculated as 0.942. In addition, the Bartlett's test of sphericity result (*χ* ^2^ = 4398.160, df = 253, *p* < 0.001) confirmed that the correlations between the items were suitable for factor analysis (Table 2).

**TABLE 2:** Exploratory factor analysis (EFA) results.

Items	Factors	
	1 (Empowering)	2 (Limiting)
M24	0.888	
M23	0.846	
M21	0.770	
M26	0.754	
M25	0.753	
M17	0.753	
M27	0.733	
M22	0.716	
M18	0.713	
M20	0.707	
M16	0.680	
M14	0.633	
M15	0.596	
M2	0.577	
M28	0.522	
M29	0.459	
M9		0.915
M8		0.798
M11		0.726
M7		0.704
M10		0.679
M5		0.624
M3		0.548
M13		0.544
Eigenvalues	11.183	2.107
Explained Variance (%)	48.60	9.20
Explained Total Variance (%)	57.80	
	KMO	0.942
	*χ* ^2^	4398.160
Bartlett test	df	253
	*p*	< 0.001

*Note:* Extraction method: principal axis factoring. Rotation method: Promax with Kaiser Normalization. a. Rotation converged in 3 iterations.

Abbreviations: df, degrees of freedom; EFA, Exploratory Factor Analysis.

In the exploratory factor analysis (EFA), the Promax rotation with Kaiser normalization was used, yielding a two‐factor structure. In line with commonly recommended criteria for item retention in factor analysis (Hair et al. 2019), items with low communality values and/or problematic factor loadings were removed from the scale. Accordingly, item 4 showed cross‐loadings, items 6 and 12 did not load saliently on any factor, and items 1, 19, 30, 31, 32, 33, 34, and 35 exhibited low communalities (< 0.40) and/or low factor loadings (< 0.40). In total, 11 items were excluded from the scale. In the two‐factor structure that emerged from the analysis, the first factor (Empowering) comprises 16 items, and the second factor (Limiting) comprises eight items. This two‐factor structure explains 57.8% of the total variance (Table 2).

**FIGURE 1:**
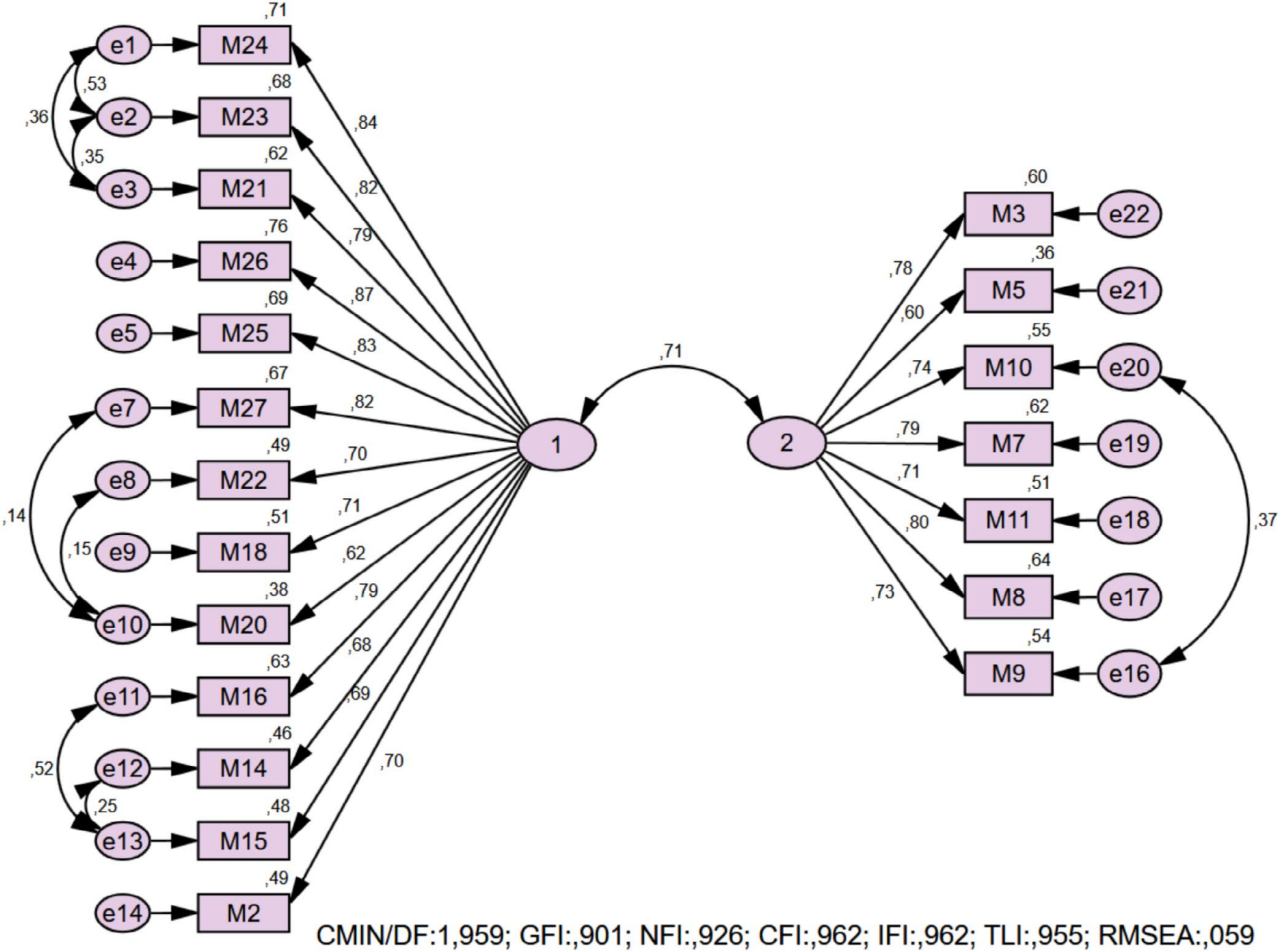
Confirmatory factor analysis.

Confirmatory Factor Analysis (CFA) was conducted to verify the two‐factor structure obtained from the EFA. The model refinement process was carried out iteratively to ensure both statistical robustness and theoretical coherence. As shown in Table 3, the initial two‐factor model comprising 24 items yielded fit indices below acceptable thresholds (*χ* ^2^/df = 3.535, GFI = 0.784, CFI = 0.881, TLI = 0.869, NFI = 0.842, IFI = 0.882, RMSEA = 0.095, SRMR = 0.053). Examination of the modification indices revealed that items 13, 17, 28, and 29 exhibited high error covariances with multiple items. It was determined that the model fit would likely not reach acceptable levels; therefore, they were removed from the analysis. Following the removal of these items, the error covariances of the remaining 20 items were re‐examined. Some item pairs continued to exhibit elevated error covariances, interpreted as residual semantic overlap between these items. To optimize model fit, error covariance adjustments were introduced between specific item pairs within the same factor (e1–e2, e10–e8, e16–e20), as illustrated in Figure 1. These modifications were applied conservatively, and no error covariances were specified across factors. As a result of these adjustments, meaningful improvements in fit indices were achieved for the final 20‐item model. All fit indices for the final model exceeded the commonly accepted thresholds: *χ* ^2^/df = 1.959, GFI = 0.901, CFI = 0.962, TLI = 0.955, NFI = 0.926, IFI = 0.962, RMSEA = 0.059, and SRMR = 0.040 (Table 3).

**TABLE 3:** Comparison of fit indices for the initial and final CFA models.

	*χ* ^2^/df	GFI	CFI	TLI	NFI	IFI	RMSEA	SRMR
Acceptable Thresholds	< 3.00	≥ 0.900	≥ 0.900	≥ 0.900	≥ 0.900	≥ 0.900	< 0.080	< 0.050
Initial Model (24 items)	3.535	0.784	0.881	0.869	0.842	0.882	0.095	0.053
Final Model (20 items)	1.959	0.901	0.962	0.955	0.926	0.962	0.059	0.040

Abbreviations: CFA, Confirmatory Factor Analysis; CFI, Comparative Fit Index; df, Degrees of freedom; GFI, Goodness of Fit Index; IFI, Incremental Fit Index; NFI, Normed Fit Index; RMSEA, Root Mean Square Error of Approximation; SRMR, Standardized Root Mean Square Residual (Hair et al. 2019; Kalkbrenner 2021); TLI, Tucker‐Lewis Index; *χ* ^2^, Chi‐square.

The CFA results, along with the composite reliability (CR) and average variance extracted (AVE) values for the 20‐item version of the scale, are presented in Table 4. According to these results, the scale items' factor loadings ranged from 0.598 to 0.869. For Factor 1, the CR and AVE values were 0.947 and 0.581, respectively; for Factor 2, they were 0.893 and 0.546, respectively (Table 4).

**TABLE 4:** Confirmatory factor analysis, convergent validity, and reliability.

Factors	Items	CFA results (*n* = 280)					Reliability results			
		*λ*	*λ* ^2^	1 − *λ* ^2^	AVE	CR	CITC	*α*	SB	GSH
Empowering	M2	0.700	0.489	0.511	0.581	0.947	0.621	0.943	0.935	0.934
	M14	0.681	0.464	0.536			0.687			
	M15	0.694	0.482	0.518			0.709			
	M16	0.795	0.632	0.368			0.766			
	M18	0.714	0.510	0.490			0.697			
	M20	0.616	0.380	0.620			0.575			
	M21	0.787	0.620	0.380			0.765			
	M22	0.697	0.486	0.514			0.656			
	M23	0.823	0.677	0.323			0.800			
	M24	0.841	0.707	0.293			0.827			
	M25	0.833	0.694	0.306			0.787			
	M26	0.869	0.755	0.245			0.804			
	M27	0.818	0.668	0.332			0.776			
Limiting	M3	0.777	0.604	0.396	0.546	0.893	0.690	0.883	0.870	0.837
	M5	0.598	0.358	0.642			0.555			
	M7	0.786	0.618	0.382			0.704			
	M8	0.799	0.639	0.361			0.703			
	M9	0.734	0.539	0.461			0.726			
	M10	0.744	0.553	0.447			0.698			
	M11	0.714	0.510	0.490			0.671			

Abbreviations: AVE, Average Variance Extracted; CFA, Confirmatory Factor Analysis; CITC, Corrected Item‐Total Correlation; CR, Composite Reliability Coefficient; GSH, Guttman Split‐Half coefficient; SB, Spearman‐Brown coefficient; *α* (Alpha), Cronbach's alpha coefficient; *λ*, Factor loading.

### 3.4 Reliability Analysis

When performing reliability analyses of the scale, internal consistency and split‐half reliability were examined. Item‐total score correlations range from 0.575 to 0.827 for Factor 1 (Empowering) and 0.555 to 0.726 for Factor 2 (Limiting). As a result of the internal consistency analysis, Cronbach's alpha values for Factors 1 and 2 were 0.943 and 0.883, respectively. In terms of split‐half reliability, the Spearman‐Brown coefficient was 0.935, and the Guttman Split‐Half coefficient was 0.934 in Factor 1; these values were 0.870 and 0.837, respectively, for Factor 2 (Table 4).

Item discrimination was evaluated by comparing the upper and lower 27% groups. In this analysis, the scores of 302 participants were ranked from highest to lowest, and the lower and upper 27% groups were formed. An independent samples *t*‐test was then used to compare the two groups. The mean score of the upper 27% group of the Empowering sub‐dimension was 48.54 ± 2.81, the mean score of the lower 27% group was 20.91 ± 6.05, and the difference between the groups was found to be statistically significant (*t* = 50.872, df = 211.805, *p* < 0.001). The mean score of the upper 27% group of the Limiting sub‐dimension was 27.68 ± 0.47, the mean score of the lower 27% group was 13.14 ± 4.18, and the difference between the groups was found to be significant (*t* = 42.463, df = 153.743, *p* < 0.001; Table 5).

**TABLE 5:** 27% Lower‐upper group comparison results of the Scale to Assess Empowering Nurse Leader Communication Behaviours (*n* = 302).

Scale sub‐dimensions	Sub‐groups	*n*	$\bar{x}$ ± SD	*t*	df	*p*
Empowering	Upper 27%	151	48.54 ± 2.81	50.872	211.805	**< 0.001**
	Lower 27%	151	20.91 ± 6.05			
Limiting	Upper 27%	151	27.68 ± 0.47	42.463	153.743	**< 0.001**
	Lower 27%	151	13.14 ± 4.18			

*Note:* Bold values indicate statistical significance at *p* < 0.001.

Abbreviations: $\bar{x}$, Mean; df, degrees of freedom; *n*, sample size; SD, standard deviation; *t*, independent samples *t* value.

A test–retest reliability analysis was performed to assess the scale's invariance over time. As a result of that analysis, the intraclass correlation coefficient (ICC) was calculated as 0.868 (*p* < 0.001) for the Empowering sub‐dimension and 0.932 (*p* < 0.001) for the Limiting sub‐dimension.

## 4 Discussion

This methodological study was conducted to adapt the scale developed to assess nurse leader empowering communication behaviours to the Turkish culture and to examine its psychometric characteristics. The evaluations confirmed the scale's language validity. The content validity index (CVI) of 0.833 indicates that the scale exceeds the acceptable level (> 0.80) specified in the literature and that the scale items are sufficient to measure the targeted concept (Alpar 2022).

In the analysis evaluating construct validity, the KMO value (> 0.70) and the Bartlett test (*p* < 0.001) indicated that the data were suitable for factor analysis (George and Mallery 2024). The EFA results revealed a two‐factor structure. Consistent with the original scale, the first factor was named “Empowering” and the second factor “Limiting”. These two factors explain 57.80% of the total variance. In the literature, an explained variance of 40% or higher is considered sufficient for scale development and adaptation studies (Büyüköztürk 2024). This two‐factor structure is generally consistent with the conceptual framework proposed by Hopkinson et al. (2021), who identified empowering and limiting communication as the core dimensions of the scale. In cross‐cultural adaptations, factor structures should be evaluated using both EFA and CFA (Gronier 2022), and psychometric properties should be tested while accounting for potential linguistic and cultural differences. During EFA, items were retained using a factor loading criterion of ≥ 0.40 and a communality criterion of ≥ 0.40. This criterion was chosen based on Hair et al.'s (2019) suggestion that appropriate minimum loading cut‐offs should be tailored to the sample size and specific study context. Although some sources cite loadings around 0.30 as minimally acceptable, using higher thresholds can improve factor clarity and interpretability (Tabachnick and Fidell 2007; Çokluk et al. 2025). Additionally, Hair et al. (2019) note that items with low communalities may be poorly represented by the factor solution and may be removed to preserve structural integrity.

The transition from EFA to CFA maintained the scale's structural integrity. While the removal of four items and the inclusion of within‐factor error covariances were necessary to achieve adequate model fit, these modifications were applied carefully to preserve the construct's theoretical structure. The resulting 20‐item model demonstrated satisfactory psychometric properties, in line with recommended validation standards for scale adaptation studies (Hair et al. 2019; Kalkbrenner 2021).

Although the final 20‐item factor structure was similar to the original scale, some differences emerged in the component structure. The original scale consists of eight components: intelligibility, attitude, listening, openness, feedback, empathy, nonverbal communication, and paralanguage. In the Turkish adaptation, seven items representing nonverbal communication and one item representing paralanguage were removed due to low factor loadings, overlapping characteristics, and limited contribution to model fit. Consequently, the scale was reduced from eight to six components. These differences may be associated with the cultural and organizational characteristics of the institutions in which the studies were conducted. The original scale was developed in military hospitals, which are typically characterized by strict hierarchical structures and a clear chain of command. In contrast, the university hospital where the present study was conducted has a more flexible hierarchical structure. This situation may facilitate more open communication between nurses and nurse managers. According to Hofstede et al. (2010), differences in power distance may influence hierarchical communication patterns. This framework may partly explain the variations observed between institutional contexts. Overall, the findings suggest that nurses tend to perceive and report empowering or limiting communication behaviours primarily in terms of explicit and verbal dimensions. Considering cross‐cultural differences in the production and interpretation of nonverbal behaviours, individuals are generally more sensitive to cues from their own cultural group (Pang et al. 2024). Because nonverbal and paralinguistic elements are embedded in routine interactions, they may be less consciously recognised and therefore less frequently reflected in self‐report measures. Future cross‐cultural studies comparing empowering nurse leader communication behaviours across different healthcare systems and cultural contexts may further clarify these findings.

In the analysis evaluating the scale's convergent validity, the CR values for the factors exceeded 0.70, and the AVE values exceeded 0.50. These results provide evidence that the latent structures adequately explain the variance in the observed variables and support the scale's convergent validity (Hair et al. 2019). The item‐total correlation coefficients for the scale ranged from 0.555 to 0.827 (*p* < 0.001). Although coefficients above 0.30 are generally considered acceptable in the literature, some authors recommend a threshold of 0.50 for high item discrimination (Esin 2014). In the present study, all items exceeded the 0.50 threshold, indicating strong item discrimination and consistent measurement of the intended construct.

The findings from the reliability analyses provide strong evidence of both internal consistency and split‐half reliability. The Cronbach's alpha coefficients for the Empowering (*α* = 0.943) and Limiting (*α* = 0.883) sub‐dimensions demonstrate a high level of internal consistency. The alignment of these values with those reported in the original study by Hopkinson et al. (2021) (0.972 and 0.935, respectively) confirms that the scale's structural reliability is preserved within the Turkish cultural context. Furthermore, the high Spearman–Brown and Guttman split‐half coefficients provide additional support for the items' homogeneous structure and their ability to consistently and stably reflect the intended construct (Alpar 2022; DeVellis 2017).

The results of the 27% upper‐group analysis, conducted to evaluate the scale's item discrimination, revealed statistically significant differences between the groups in both the Empowering and Limiting sub‐dimensions (*p* < 0.001). The significant differences observed in both sub‐dimensions show that the scale has a strong distinguishing characteristic.

The intraclass correlation coefficient (ICC) values were 0.868 for the Empowering sub‐dimension and 0.932 for the Limiting sub‐dimension. According to Koo and Li (2016), ICC values between 0.75 and 0.90 indicate good reliability, whereas values above 0.90 indicate excellent reliability. These findings indicate that both sub‐dimensions yield stable, consistent measurements over time, with limited influence from random measurement error.

### 4.1 Limitations

The present study has some limitations. First, the conceptual framework of the Turkish version differs from that of the original scale, with six components instead of eight. In particular, the exclusion of nonverbal and paralinguistic dimensions from the construct in the Turkish version may reflect potential difficulties participants experienced in accurately recalling and quantifying implicit behaviours (e.g., tone of voice, facial expressions) during retrospective self‐report. Second, the study was conducted in a single institution, and findings may have been affected by its organizational structure, leadership style, and communication culture. Consequently, the generalizability of the results to other institutions, healthcare settings, or cultural contexts may be limited. Another limitation of the study is the use of a self‐report scale to assess communication behaviours. The use of such scales in research may lead to social desirability bias.

## 5 Conclusions

The Turkish version of the Scale to Assess Empowering Nurse Leader Communication Behaviours was confirmed as a valid and reliable scale, consisting of 20 items and two factors (Empowering and Limiting). The psychometric properties of the scale are acceptable in both content and construct validity. Reliability analyses indicated that the scale provided consistent, stable measurements over time. This scale may be used to assess the empowering communication behaviours of nurse managers in Turkish society.

### 5.1 Implications for Nursing Practice

Effective communication competencies enable leaders to build a collaborative environment, maintain patient safety standards, and achieve cost‐effective healthcare outcomes (AONE 2015). This scale facilitates assessment of the empowering and limiting communication behaviours of nurse managers. In healthcare delivery settings, the scale can be used periodically at the unit or hospital level to evaluate nurse managers' communication behaviours. Based on monitoring results, effective communication training, coaching, or mentoring programs can be designed for nurse managers in units where such needs are identified. In addition, the use of the scale in leadership development programs and performance evaluation processes can enable regular monitoring and facilitate the planning of improvement initiatives. Finally, the scale enables comparison of empowering nurse leader communication behaviours across different hospitals, regions, and cultural contexts, thereby supporting national and international comparisons. Such comparisons may inform the development of organisational strategies and broader policy recommendations to empower nurse leadership and communication across diverse healthcare systems.

## Author Contributions

All authors have agreed on the final version and meet at least one of the following criteria [recommended by the ICMJE http://www.icmje.org/recommendations/]. Made substantial contributions to conception and design, or acquisition of data or analysis and interpretation of data; Sultan Türkmen Keskin, Meltem Özduyan Kılıç, Ayten Demir. Involved in drafting the manuscript or revising it critically for important intellectual content; Sultan Türkmen Keskin, Meltem Özduyan Kılıç, Ayten Demir. Given final approval of the version to be published and participated sufficiently in the work to take public responsibility for appropriate portions of the content; Sultan Türkmen Keskin, Meltem Özduyan Kılıç, Ayten Demir. Agreed to be accountable for all aspects of the work in ensuring that questions related to the accuracy or integrity of any part of the work are appropriately investigated and resolved; Sultan Türkmen Keskin, Meltem Özduyan Kılıç, Ayten Demir.

## Funding

The authors have nothing to report.

## Disclosure

Statistical: The authors have checked to make sure that our submission conforms to the Journal's statistical guidelines described here. The statistics were checked prior to submission by an expert statistician (Hakan Dokumuş, E‐mail: dokumushakan@gmail.com). The author(s) affirm that the methods used in the data analyses are suitably applied to their data within their study design and context, and the statistical findings have been implemented and interpreted correctly.

## Ethics Statement

Approval was obtained from the Ankara University Ethics Committee (Date: September 13, 2023; number: E‐64446339‐020‐154252).

## Conflicts of Interest

The authors declare no conflicts of interest.

## Data Availability

The data that support the findings of this study are available from the corresponding author upon reasonable request.
